# BMP signaling components in embryonic transcriptomes of the hover fly *Episyrphus balteatus *(Syrphidae)

**DOI:** 10.1186/1471-2164-12-278

**Published:** 2011-05-31

**Authors:** Steffen Lemke, Dionysios A Antonopoulos, Folker Meyer, Marc H Domanus, Urs Schmidt-Ott

**Affiliations:** 1 University of Chicago, Dept. of Organismal Biology and Anatomy, CLSC 921B, 920 E. 58th Street, Chicago, IL 60637, USA; 2 Current Address: University of Heidelberg, Centre for Organismal Studies, Im Neuenheimer Feld 230, 69120 Heidelberg, Germany; 3 Argonne National Laboratory, Institute for Genomics & Systems Biology, 9700 S. Cass Avenue, Argonne, IL 60439, USA

## Abstract

**Background:**

In animals, signaling of Bone Morphogenetic Proteins (BMPs) is essential for dorsoventral (DV) patterning of the embryo, but how BMP signaling evolved with changes in embryonic DV differentiation is largely unclear. Based on the extensive knowledge of BMP signaling in *Drosophila melanogaster*, the morphological diversity of extraembryonic tissues in different fly species provides a comparative system to address this question. The closest relatives of *D. melanogaster *with clearly distinct DV differentiation are hover flies (Diptera: Syrphidae). The syrphid *Episyrphus balteatus *is a commercial bio-agent against aphids and has been established as a model organism for developmental studies and chemical ecology. The dorsal blastoderm of *E. balteatus *gives rise to two extraembryonic tissues (serosa and amnion), whereas in *D. melanogaster*, the dorsal blastoderm differentiates into a single extraembryonic epithelium (amnioserosa). Recent studies indicate that several BMP signaling components of *D. melanogaster*, including the BMP ligand Screw (Scw) and other extracellular regulators, evolved in the dipteran lineage through gene duplication and functional divergence. These findings raise the question of whether the complement of BMP signaling components changed with the origin of the amnioserosa.

**Results:**

To search for BMP signaling components in *E. balteatus*, we generated and analyzed transcriptomes of freshly laid eggs (0-30 minutes) and late blastoderm to early germband extension stages (3-6 hours) using Roche/454 sequencing. We identified putative *E. balteatus *orthologues of 43% of all annotated *D. melanogaster *genes, including the genes of all BMP ligands and other BMP signaling components.

**Conclusion:**

The diversification of several BMP signaling components in the dipteran linage of *D. melanogaster *preceded the origin of the amnioserosa.

[Transcriptome sequence data from this study have been deposited at the NCBI Sequence Read Archive (SRP005289); individually assembled sequences have been deposited at GenBank (JN006969-JN006986).]

## Background

Across animals, the Bone Morphogenetic Protein (BMP) signaling pathway plays a major role in specifying the dorsoventral (DV) axis [1,2]. However, the components of the BMP pathway have been repeatedly modified through lineage specific gene duplications and gene losses [3,4]. Whether some of these genetic changes correlate with the origin of species-specific morphological traits that develop under the control of the BMP pathway is unknown. Flies (Diptera) provide an excellent opportunity to address this question firstly because the BMP signaling pathway of *Drosophila melanogaster *has been studied in great detail [5,6], and secondly because tissue specification presumably under the control of BMP signaling along the DV axis of dipterans has undergone significant change [7]. In *D. melanogaster*, dorsal blastoderm differentiates into a single extraembryonic epithelium, called amnioserosa, which closes the developing embryo dorsally [8]. This tissue is found in higher cyclorrhaphan flies (Schizophora), but in other dipterans, dorsal blastoderm gives rise to distinct serosal and amniotic epithelia [9-11]. Serosa and amnion develop from an amnioserosal fold at the margins of the gastrulating embryo. The outer cell layer of this fold becomes the serosa, which closes about the embryo. Its inner cell layer detaches from the serosa but retains continuity with the embryo while closing dorsally (lower cyclorrhaphan flies) or ventrally (non-cyclorrhaphan dipterans). The lower cyclorrhaphan syrphids represent the closest relatives of *D. melanogaster *that have been shown to develop distinct serosa and amnion tissues [9]. Therefore, they are of particular interest in efforts to understand how the origin of the amnioserosa as a new morphology is linked to changes in the underlying developmental gene network.

In previous studies we have characterized the role that the homeobox gene *zerknüllt *(*zen*) may have played in the origin of amnioserosa development [reviewed in 7]. The transcription factor Zen is regulated by BMP signaling and essential in serosa specification in non-schizophoran insects and amnioserosa specification in *D. melanogaster *[9,12-14]. In lower Cyclorrhapha and more distant relatives of *D. melanogaster, zen *expression in the serosa is maintained after gastrulation, i. e., when the serosa begins to spread over the embryo [9], whereas in *D. melanogaster zen *expression in the amnioserosa is down-regulated immediately after gastrulation [13]. In lower cyclorrhaphan flies, postgastrular down-regulation of *zen *abrogates serosa development and results in the formation of a single extraembryonic tissue with amniotic gene expression [15]. Thus, the repression of this single transcription factor may account for the morphological tissue reorganization that accompanied the origin of the amnioserosa. However, loss of postgastrular *zen *expression does not explain, why in lower cyclorrhaphan and non-cyclorrhaphan dipterans the patterning of the dorsal blastoderm results in the specification of two distinct extraembryonic tissues types as opposed to one in schizophoran (i. e. higher cyclorrhaphan) flies such as *D. melanogaster*.

In *D. melanogaster*, amnioserosa specification occurs at the dorsal midline and requires peak-levels of BMP activity [14,16], which are provided through the interaction of two extracellular ligands, Dpp and Scw [17-19]. Both ligands are secreted into the perivitelline space and transported towards the dorsal midline [17,20-24], where BMP-ligand dimers are released from antagonists to activate a receptor complex and initiate intracellular signaling [17,20,25-27]. A cell autonomous autoregulatory loop further increases the BMP signal at the dorsal midline and generates a narrow and sharply delineated domain of BMP peak activity [20,28]. Dpp is essential for BMP activity and controls the specification of all tissues that develop under the control of this pathway in the early embryo, including amnioserosa and dorsal ectoderm [16,29]. Scw boosts BMP activity along the dorsal midline and is in particular required for amnioserosa specification [19].

In other dipterans, the molecular mechanisms that specify the amnion and serosa are not known. Expression studies in a mosquito suggest that a tighter expression of the Dpp antagonist short gastrulation (Sog) leads to broader BMP signaling, which in turn may allow for the specification of two versus one extraembryonic tissue type [11]. Additionally, Scw is absent from the genomes of mosquitoes and other insects, and it has been suggested that its origin may correlate with the origin of the amnioserosa [3]. Several other BMP signaling components of *D. melanogaster *resulted from gene duplications that have been mapped to the dipteran lineage, while others - known from the BMP pathways of vertebrates - were lost in the lineage leading to *D. melanogaster *[4]. Here we use embryonic transcriptome data of the hover fly *Episyrphus balteatus *(Syrphidae) to address the question of whether evolutionary changes in the complement of BMP signaling components occurred in correlation with the origin of the amnioserosa. Specifically, we found that with the possible exception of one gene-duplication (*crossveinless*/*shrew*) and one gene loss (DAN), genes encoding known BMP signaling components, including *scw*, are conserved across the schizophoran boundary of the dipteran tree. Thus, most or all of the gains and losses of BMP signaling genes in the dipteran lineage do not correlate with the origin of the amnioserosa, suggesting that the origin of amnioserosa specification was probably achieved by rearranging the interaction of established factors.

## Results and Discussion

### Putative Orthologues of 6013 *E. balteatus *Genes

**Figure 1:**
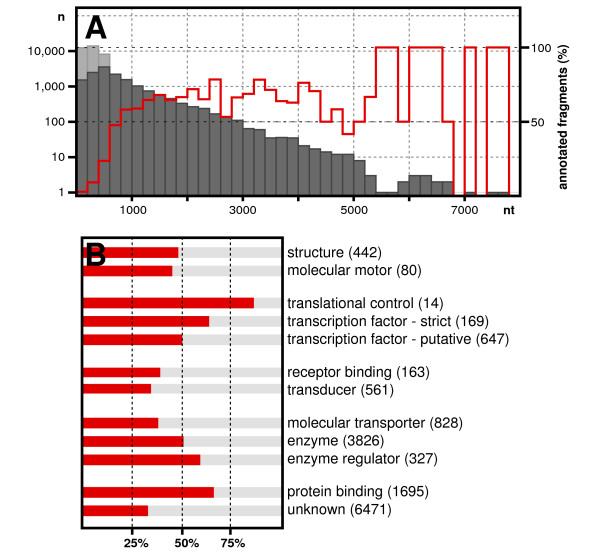
**Overview of *E. balteatus *transcriptome assembly and annotation**. (**A**) Number of sequences per 200-nucleotide-bin (y-axis) is shown as function of assembled sequence lengths (x-axis). Numbers of assembled contigs (dark grey) and singletons (light grey) are shown on a logarithmic scale (left scale), and the proportion of annotated *E. balteatus *sequences (red graph) are shown in percent (right scale). Note that sequences below 100 nt were not used for further analyses and were excluded from bin 0-200. (**B**) Gene ontology assignments of annotated *E. balteatus *genes, red bars indicate the proportion of annotated *E. balteatus *sequences in percent.

We sequenced the transcriptome of *E. balteatus *embryos at two successive time points during early embryogenesis: 0-0.5 hrs old embryos to sample pre-blastoderm stages prior to the onset of zygotic transcription ("maternal library"), and 3-6 hrs old embryos to sample blastoderm and gastrulation stages after the onset of zygotic transcription ("zygotic library"). The cDNA libraries that we prepared from these developmental stages were normalized (Additional file 1A,B) and sequenced using the 454 GS FLX Titanium platform. Following removal of contaminants (see Material and Methods, Additional file 1C, D) reads from both libraries were pooled and assembled using the Newbler Assembler from Roche. Above our chosen cutoff of 100 nt, this assembly yielded a total of 16,950 contigs with an average length of 798 nt (13.5 MB) and 26,862 singletons with an average length of 264 nt (7.1 MB). This data set (20.6 MB total sequence data) was used in subsequent analyses (Figure 1A).

To identify *E. balteatus *genes, we pooled sequence data from both libraries and performed reciprocal BLAST against annotated genes of *D. melanogaster*. Based on reciprocal hits, we identified putative *D. melanogaster *orthologues of 6013 *E. balteatus *genes. In total, about 8.1 MB (39%) of the assembled *E. balteatus *sequence data could be annotated (red line in Figure 1A), corresponding to 43% of annotated *D. melanogaster *genes. Specifically, we recovered 85% of genes annotated for translational control, over 60% of the genes known to encode gene-specific transcription factors (transcription factor - strict [30]), 50% of the genes associated with known and putative gene-specific transcription factors (transcription factor - putative [30]), about 50% of genes associated with structural functions (structure) and enzymes, and 30-40% of genes associated with receptor binding, molecular transporters, and signal transduction (transducer) (Figure 1B).

### Assessment of Coverage

To estimate the coverage of developmental genes, we separately mapped the reads from each library back onto all of 14 previously described *E. balteatus *segmentation genes, which comprise orthologues of *bicoid *(*Eba-bcd*), *caudal *(*Eba-cad*), *nanos *(*Eba-nos*), *torso *(*Eba-tor*), *orthodenticle *(*Eba-otd*), *hunchback *(*Eba-hb*), *Krüppel *(*Eba-Kr*), *knirps *(*Eba-kni*), *giant *(*Eba-gt*), *hairy *(*Eba-h*), *even-skipped *(*Eba-eve*), *zerknüllt *(Eba-*zen*), *tailless *(*Eba-tll*), and *huckebein *(*Eba-hkb*) [9,31-33]. The combined maternal and zygotic coverage of all fourteen genes was on average 8.6-fold (Additional file 2), slightly less than the average coverage of the entire assembled transcriptome (~12-fold). However, coverage of 5'UTR sequences (2.0-fold) and 3'UTR sequences (1.6-fold) was considerably lower than the coverage of ORF sequences (12.5-fold). As the CG content of UTRs (20%) was notably lower than the CG content of the ORFs (43%), a systematic bias against AT rich sequences may have been introduced by less efficient annealing of the random hexamer primers during first strand cDNA synthesis. In any case, coverage of our *E. balteatus *transcriptome data set was high enough to identify at least fragments of genes known to be active during early embryonic development.

**Figure 2:**
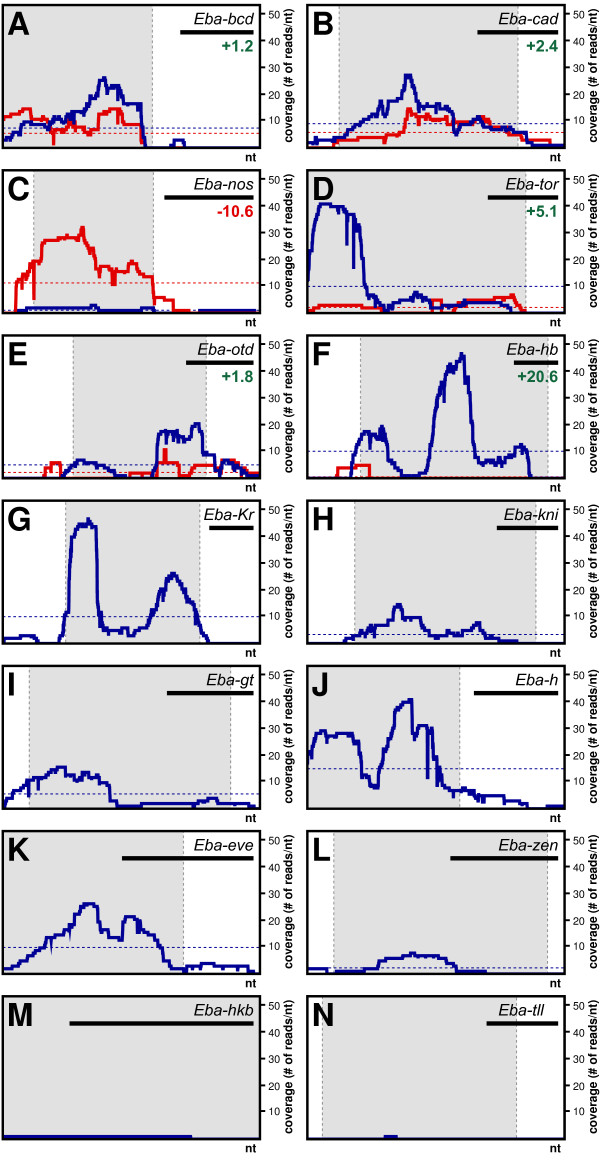
**Coverage of previously identified *E. balteatus *developmental genes**. (**A**) *Eba-bcd *(GenBank accession: HM044914), (**B**) *Eba-cad *(FJ387230), (**C**) *Eba-nos *(FJ387226), (**D**) *Eba-tor *(HM044920), (**E**) *Eba-otd *(FJ387225), (**F**) *Eba-hb *(FJ387229), (**G**) *Eba-Kr *(HM044918), (**H**) *Eba-kni *(HM044916), (**I**) *Eba-gt *(HM044915), (**J**) *Eba-h *(AY645032), (**K**) *Eba-eve *(AY645031), (**L**) Eba-*zen *(DQ323932), (**M**) Eba-kkb (HM067828), and (**N**) *Eba-tll *(HM044919). Coverage (i.e., number of reads) is shown as function of nucleotide position in cDNA (x-axis; ORF in grey). Coverage in the maternal cDNA pool (0-0.5 hrs) is labeled in red, coverage in the zygotic cDNA pool (3-6 hrs) is labeled in blue. Average coverage is indicated by dotted horizontal lines. Fold-changes in coverage levels from maternal to zygotic pool are given in green (increase) or red (decrease) numbers. A listing of coverage values detailing ORF and UTR is given in Additional file 2. Scale bars (top right of panel) are 500 nt.

All fourteen genes were represented with at least one read from the zygotic library (blue lines in Figure 2A-N), which is consistent with our previous finding that all these genes are expressed in the 3-6 hours time window of embryonic development [9,31-33]. For six of these genes (*Eba-bcd, Eba-cad, Eba-nos, Eba-tor, Eba-otd, Eba-hb*) we also obtained reads from the maternal library (red lines in Figure 2A-F). Previous and new (Additional file 3A-C) in situ hybridization data indicated maternal expression of *Eba-bcd, Eba-cad, Eba-nos, Eba-tor *and *Eba-otd*, but not of *Eba-hb*. Quantitative PCR (qPCR) on non-normalized cDNA suggested an 18-fold increase of *Eba-hb *expression levels following the onset of zygotic transcription, whereas expression levels both of *Eba-otd *and *Eba-cad *increased by about 2-fold (orange bars in Additional file 3D). Coverage of these genes in the maternal and zygotic transcriptomes closely reflected our qPCR data (light grey bars in Additional file 3D), suggesting that, despite cDNA normalization, the coverage of these genes remained roughly proportional to their expression levels.

### BMP Signaling Components in the *E. balteatus *Transcriptome Database

In *D. melanogaster*, BMP signaling at the dorsal side of the blastoderm is required to specify the amnioserosa as a single extraembryonic tissue (see Background). Based on mosquito data, it has been suggested that in lower dipterans restricted expression of the BMP antagonist Short gastrulation (Sog) may account for an expanded BMP signaling domain in the dorsal blastoderm, which resolves into serosa and amnion territories [11]. Furthermore, it has been suggested that the complement of BMP signaling components changed in the dipteran lineage in parallel with the origin of the amnioserosa [3]. We used our transcriptome database as a tool to test the latter idea by searching for *E. balteatus *homologues of specific BMP signaling components of *D. melanogaster*.

**Figure 3:**
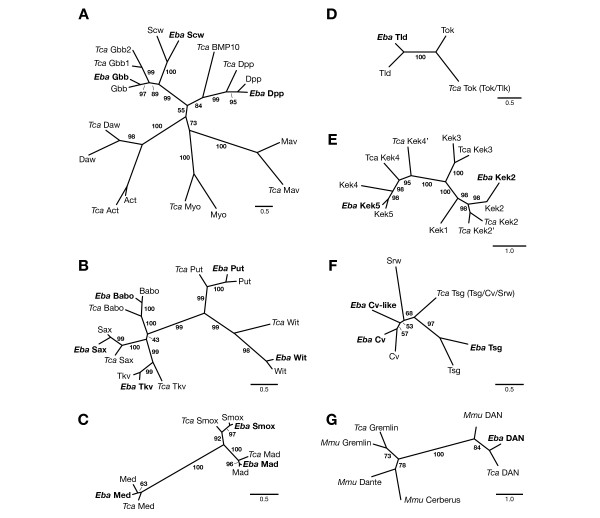
**Orthologies of BMP signaling components based on maximum likelihood gene trees using predicted amino acid sequences**. (**A**) TGFß ligands (substitution model DCMut+i+g+g); (**B**) TGFβ type I and type II receptors (substitution model WAG+i+g+f); (**C**) SMAD family (substitution model LG+g+f); (**D**) Metalloproteases related to Tld and Tok (substitution model Dayhoff+g+f); (**E**) Kekkon family (substitution model LG+i+g+f); (**F**) Crossveinless family (substitution model Dayhoff+i+g+f); (**G**) DAN family (substitution model Dayhoff+i+g+f); scale bars indicate estimated changes/position. BeetleBase (*T. castaneum*) and GenBank accession numbers (all others): (A) Act (Activin-β; NM_143685), Tca Act (TC015806), Tca BMP10 (TC006506), Daw (dawdle; NM_078737), Tca Daw (TC04297), Dpp (decapentaplegic; NM_057963), Eba Dpp (JN006972), Tca Dpp (TC008466), Gbb (glass bottom boat; NM_057992), Eba Gbb (JN006973), Tca Gbb1 (TC014017), Tca Gbb2 (TC014018), Mav (maverick; NM_079887.2), Tca Mav (TC004299), Myo (myoglianin; NM_166786) Tca Myo (TC015805), Scw (screw; NM_080124.4), Eba Scw (JN006978); (B) Babo (baboon; NM_057652), Eba Babo (JN006969), Tca Babo (TC003240), Put (punt; NM_169591), Eba Put (JN006976), Tca Put (TC011357), Sax (saxophone; NM_078928), Eba Sax (JN006977), Tca Sax (TC015984), Tkv (thickveins; NM_175975), Eba Tkv (JN006980), Tca Tkv (TC006474), Wit (wishful thinking; NM_079953), Eba Wit (JN006986), Tca Wit (TC009314); (C) Mad (mothers against dpp; NM_057669), Eba Mad (JN006974), Tca Mad (TC014924), Med (Medea; NM_079871), Eba Med (JN006975), Tca Med (TC010848), Smox (smad on X; NM_078524), Eba Smox (JN006979), Tca Smox (TC010162); (D) Tld (tolloid; NM_079763), Eba Tld (JN006985), Tok (tolkin; NM_057531), Tca Tok (TC011197); (E) Kek1 (kekkon-1; NM_078835), Kek2 (NM_078827), Tca Kek2 (TC007053), Tca Kek2' (TC008448), Eba Kek2 (JN006983), Kek3 (NM_078851), Tca Kek3 (TC007226), Kek4 (NM_135771), Tca Kek4 (TC007110), Tca Kek4' (TC008070), Kek5 (NM_133154), Eba Kek5 (JN006984); (F) Cv (crossveinless; NM_080525), Eba Cv (JN006982), Eba Cv-like (JN006970), Srw (shrew; NM_139629), Tsg (twisted gastrulation; NM_078580), Eba Tsg (JN006981), Tca Tsg (TC003620); (G) Mmu Cerberus (Mus musculus; NM_009887), Mmu Dante (NM_201227), Mmu Gremlin (NM_011824), Tca Gremlin (TC007044), Eba DAN (JN006971), Mmu DAN (NM_008675.2), Tca DAN (TC014861).

Specific BMP signaling components include (1) extracellular ligands, (2) transmembrane receptors, (3) intracellular signal transducers, and (4) extracellular modulators of ligands. The *D. melanogaster *genome contains a total of three genes encoding BMP ligands: *decapentaplegic *(*dpp*) [18], *glass bottom boat *(*gbb*) [34], and *screw *(*scw*) [19]. These are selectively used depending on the developmental context [5]. In other insects, only homologues of *dpp *and *gbb *have been found [3,4]. We identified *E. balteatus *homologues of all three ligands (Figure 3A), indicating that these genes existed prior to the origin of the amnioserosa. Consistent with previous reports [3,4], our gene tree supports a sister gene relationship between *scw *and *gbb*. As expected based on a comprehensive survey of TGF-β signaling components in the beetle *Tribolium castaneum *[4], we also identified *E. balteatus *homologues for each of the *D. melanogaster *TGF-β receptors Thickveins (Tkv) and Saxophone (Sax) [35-38], Punt (Put) [39,40], Baboon (Babo) [41], and Wishful thinking (Wit) [42,43], and the SMAD transducers Mothers against Dpp (Mad), Medea, and Smox [44-48], as well as other TGF-ß signaling components (Figure 3B,C; Additional file 4).

In *D. melanogaster*, activity of BMP ligands is modulated by Sog [22,23,26], which in turn is regulated by the related metalloproteases Tolloid (Tld) and Tolkin (Tok) [25,49,50]. We identified *E. balteatus *homologues of *sog *as well as of *tld*. While we were not able to identify an orthologue of tok, the presence of a distinct *tld *orthologue in *E. balteatus *suggests that the dipteran gene duplication giving rise to *tld *and *tok *occurred before the origin of the amnioserosa (Figure 3D). BMP ligand activity in *D. melanogaster *is additionally modulated by Twisted gastrulation (Tsg) [51,52], Crossveinless (Cv) [53,54], Shrew (Srw) [26,55], as well as the membrane associated factors Crossveinless-2 (Cv-2) [56,57], Kekkon 5 (Kek5) [58], Pentagone (Pent) [59] and Larval Translucida (Ltl) [60]. We identified *E. balteatus *homologues of *cv-2, kek5 *(Figure 3E) as well as *tsg, cv *and an additional *cv *paralogue, *Eba-cv-like *(Figure 3F). Previous studies have suggested that *tsg, cv*, and *srw *originated by two successive duplications of a *cv*-like ancestor in the dipteran lineage [3]. Our gene tree analysis is consistent with this idea, but does not resolve whether *Eba-cv-like *is orthologous to *srw*, or whether it is the product of an independent gene duplication in *E. balteatus*. We did not identify orthologues of *pent *and *ltl *in *E. balteatus*, but putative orthologues of both genes are present in the genome of *T. castaneum *(data not shown). Thus, all currently known modulators of BMP ligand activity in *D. melanogaster *may have existed prior to the origin of the amnioserosa.

Putative orthologues of the vertebrate BMP ligands BMP10 [61] and Anti-Dorsalizing Morphogenetic Protein (ADMP) [62-64], as well as the vertebrate BMP inhibitors BAMBI [65], DAN and Gremlin [66] have been found in beetles and/or wasps but not in *D. melanogaster *[4]. Among these, we were able to identify a putative orthologue of *DAN *in *E. balteatus *(Figure 3G). The loss of this gene may correlate with the origin of the amnioserosa. However, the function of *DAN *in insects remains unknown and its potential role in BMP signaling is therefore speculative.

### Differences in maternal expression of BMP signaling components in *D. melanogaster *and *E. balteatus*

**Figure 4:**
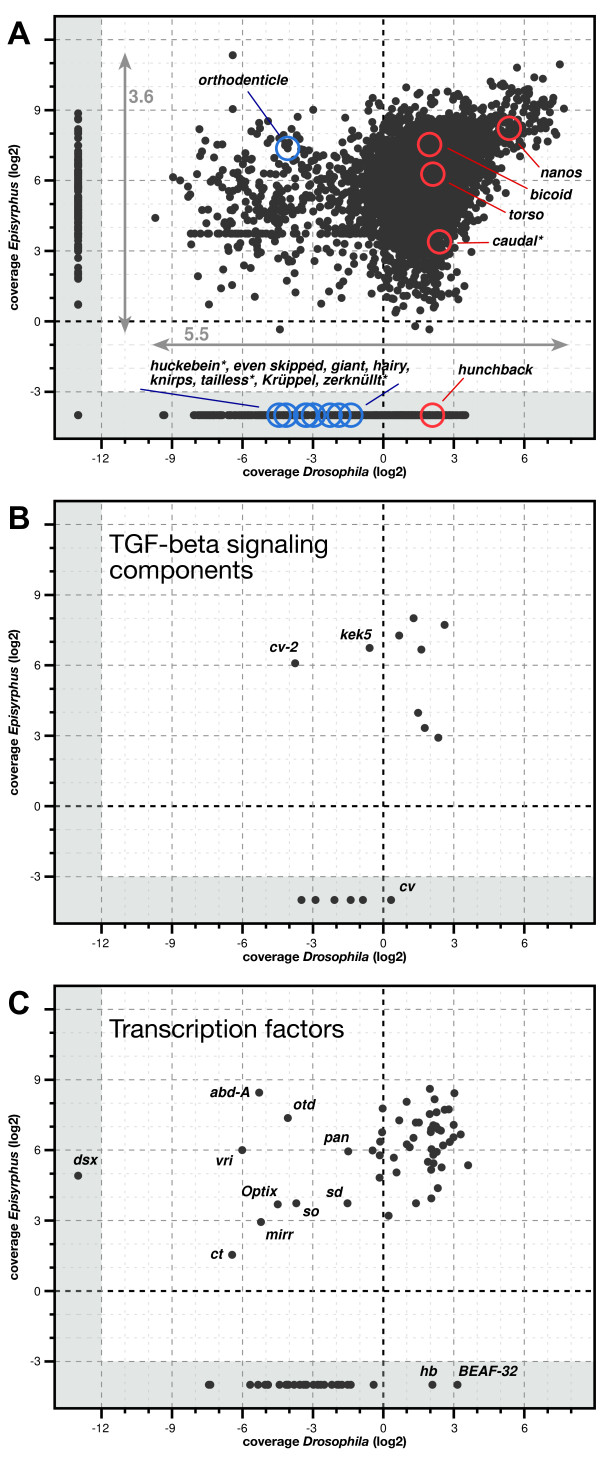
**Coverage of maternal *E. balteatus *genes in comparison with transcriptome data from 0-2 hours old *D. melanogaster *embryos**. (**A**) Scatter plot of orthologous genes for which we determined expression levels in *E. balteatus *(using 454 data from 0-0.5 hrs old embryos) and *D. melanogaster *(using RNAseq data from 0-2 hrs old embryos) on a log2 scale. The 14 previously identified *E. balteatus *genes are circled in red (if their *D. melanogaster *orthologues are known to have a maternal effect) or in blue (if their *D. melanogaster *orthologues are thought to lack a maternal effect). Asterisks demark genes that have been manually annotated. Genes not detected at the indicated developmental stage in one of the two species are plotted outside the scale (grey area). (**B,C**) Same as in (A) but limited to 15 identified genes encoding BMP signaling components (B) or 89 genes of gene specific transcription factors (C, list of genes based on flyTF, see Material and Methods). Genes that might lack significant maternal expression in one of the two species are indicated. Our analysis suggests that the zygotic *D. melanogaster *genes crossveinless-2, kekkon-5, *doublesex, abdominal-A, vrille *and *Optix *are expressed maternally in *E. balteatus*, while the maternal *D. melanogaster *transcription factor BEAF-32 may lack significant maternal expression in *E. balteatus*.

Based on our finding that coverage levels of segmentation genes in the two sequenced transcriptomes were roughly proportional to their expected expression levels (see above; Additional file 3D), we decided to globally compare maternal gene expression between *E. balteatus *and *D. melanogaster*. For this purpose, we approximated the maternal expression profiles of all annotated *E. balteatus *genes based on their coverage in the maternal transcriptome and compared them to maternal expression profiles of their *D. melanogaster *orthologues. Maternal coverage levels of all annotated *E. balteatus *genes were corrected for sequence lengths and sequencing depth (see Methods) and plotted against coverage levels of their *D. melanogaster *orthologues, which were estimated from available SOLiD total RNA sequencing data of 0-2 hour old embryos (i.e. stages when the zygotic transcriptome is still essentially silent) [67] (Figure 4). Coverage levels derived from the non-normalized *D. melanogaster *transcriptome spread by 5.5 orders of magnitude, while those of the normalized *E. balteatus *transcriptome spread by 3.6 orders of magnitude. A reduced breadth in coverage levels of *E. balteatus *genes was expected due to the normalization protocol. The coverage levels of the maternal genes *nanos, bicoid, torso*, and *caudal *were higher than 1 in the transcriptome of *D. melanogaster *and *E. balteatus*. In contrast, the zygotic genes *huckebein, even skipped, giant, hairy, knirps, tailless, Krüppel*, and *zerknüllt *showed coverage levels lower than 1 in both species (Figure 4A). Notably, the scatter plot correctly revealed the expression differences of *hunchback *(maternally expressed in *D. melanogaster *but not in *E. balteatus*) and *orthodenticle *(maternally expressed in *E. balteatus *but not in *D. melanogaster*). When restricting the data set to BMP components (Figure 4B) or transcription factors (Figure 4C), we readily identified additional candidate maternal expression differences in both gene groups. For example, the data suggest maternal expression of *crossveinless-2 *or *kekkon-5 *in *E. balteatus *but not in *D. melanogaster*, which might reflect different interactions of BMP signaling molecules and regulators in both species during early embryonic development.

## Conclusions

**Figure 5:**
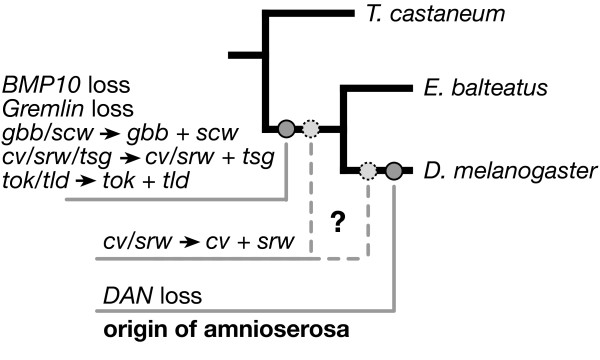
**Evolution of BMP signaling components and amnioserosa origin**.

Comprising orthologous sequences of nearly half (43%) of all annotated *D. melanogaster *genes, the newly generated transcriptome data of *E. balteatus *provide a convenient tool to identify putative orthologues of conserved insect genes. Here we used the transcriptome data of *E. balteatus *to show that the novel dipteran BMP ligand Scw and other BMP signaling components of *D. melanogaster *existed prior to the origin of the amnioserosa (Figure 5). These findings suggest that the origin of amnioserosa development was accompanied by subtle changes in the expression of conserved BMP signaling components, rather than on the origin or loss of individual genes. Modification of the BMP pathway is expected to be constrained due to its multiple functions in development. However, the duplicated genes (*gbb *and *scw*; *tld *and *tok*; *cv, tsg *and *srw) *may have relaxed these constraints, because BMP activity could now be provided by non-identical sets of genes in early (blastoderm) and later developmental stages. We suspect that increasing the role of *scw, tld, tsg *and *srw *in early (blastoderm) development at the expense of *gbb, tok *and *cv *facilitated genetic accommodation of early DV patterning following the origin of the amnioserosa. Conversely, the entire complement of the duplicated BMP signaling genes might still be required for early DV patterning in lower cyclorrhaphan flies such as *E. balteatus*.

## Methods

### Preparation of Transcriptome Library

Total RNA was prepared by homogenizing embryos in Trizol (Inivtrogen), treated with DNaseI, and enriched for polyA containing transcripts using the Oligotex kit (Qiagen). First-strand cDNA was synthesized from approximately 1 μg of mRNA. Annealing of random hexamer primers (15 mM) was at 25°C for 10 minutes, cDNA was synthesized at 50°C for 1 hour and followed by inactivation of the reverse transcriptase (Superscript, Invitrogen) at 85°C for 5 minutes. Second-strand cDNA was synthesized using the first strand reaction with Klenow DNA Polymerase at 15°C for 1.5 hours, and terminated by the addition of 0.5 M EDTA, pH 8. cDNA was purified using the QIAquick MinElute Reaction Clean-up Kit (Qiagen). cDNA ends were filled in ("polished") using a mix of Klenow DNA polymerase and T4 polynucleotide kinase with dNTPs at 20°C for 30 minutes, after which the reactions were purified again using the QIAquick MinElute Reaction Clean-up Kit. "A" overhangs were created by incubating polished cDNA with 0.2 mM dATP 0.3 U/μl Klenow exo- at 37°C for 30 minutes. cDNA was purified using the QIAquick MinElute Reaction Clean-up Kit (Qiagen), ligated with a mix of AdaptorA and AdaptorB using T4 DNA ligase, and purified again. Adaptors were generated by annealing equimolar amounts of complementary oligos in 2x TNE buffer (20 mM Tris-Cl, pH 8, 0.2 mM EDTA, pH 8, 100 mM NaCl). Oligo sequences for both adaptors were adapted from 454 Sequencing Technical Bulletin No. 004-2009, ordered from Integrated DNA Technologies and HPLC purified. Amplification of the library was performed in triplicate using Platinum Taq DNA polymerase HiFi with AdapterA and AdapterB primers. AdapterA FW and AdapterB FW primers. Pooled volumes of the library were purified using the QIAquick MinElute Reaction Clean-up Kit.

### Library Normalization and Fragment Size Selection

Libraries were normalized using the TRIMMER DIRECT cDNA Normalization Kit (Evrogen) and were carried out essentially as described in the user manual. Briefly, 400 ng of each library were suspended in hybridization buffer and split into four tubes. Following 5 hr incubation at 68°C, the aliquots were treated either with 4 units, 2 units, 1 unit, or no duplex-specific nuclease (DSN). After DSN digestion, the normalized cDNA libraries were amplified by PCR. Optimal amplification within the exponential phase of the PCR was determined visually after electrophoresing different amplification runs of the non-DSN treated sample, after which all aliquots were amplified by a total of 20 cycles. The normalization efficiency was assessed by quantitative PCR (qPCR) of *hunchback *(*Eba-hb*, 5'-CTCAGCCCGAATCCAAAT/5'-GGTTGTGGGAGTTGATGTTG, amplicon 137 bp), *caudal *(*Eba-cad*, 5'-GAAAGAATACTGCACCTCCC/5'-GTCGTTCCGATAGTTGAAGC, amplicon 79 bp), and alpha-tubulin as reference (Eba-tub, 5'-TGAGGCTCGTGAGGATTT/5'-TCACCATCTCCAGAATCCA, amplicon 71 bp). Primer efficiency was estimated from a standard curve using five different template concentrations (12.5-200 ng); all analyses were run simultaneously in triplicates. For both libraries, the normalization with 4 units DSN were chosen for sequencing. Based on optimal cycle numbers for amplification and degree of normalization, these libraries were size fractionated using agarose gel electrophoresis, excising fragments at roughly 500 bp, and then purifying them using the MinElute Gel Extraction Kit (Qiagen) prior to sequencing.

### 454 Sequencing

Transcriptome library sequencing was performed on the Roche/454 Life Sciences GS-FLX platform at the Institute for Genomics and Systems Biology's (IGSB) High-Throughput Genome Analysis Core (HGAC) at Argonne National Laboratory according to the Roche GS-FLX XLR70 Titanium emPCR and amplicon sequencing protocols. Each transcriptome library was sequenced on one region of a two region GS-FLX gasket using Roche GS-FLX XLR70 titanium sequencing reagents. An emPCR titration was initially performed on each library to determine the proper bead:library copy ratio that yielded optimal clonal bead percent enrichment to be used in the final bulk XLR70 emPCR reaction.

### Sequence Assembly

Sequencing raw data was processed with gsRunProcessor (software release 2.0.00.20) using standard quality filtering and trimming as defined by the default settings. We obtained 417,735 reads with a mean length of 243 nt for the maternal time point and 406,580 reads with a mean length of 278 nt for the zygotic time point, totaling 214.6 MB of sequence data. This raw data set was contaminated with sequences of the pea aphid *Acyrthosiphon pisum*, because *E. balteatus *requires aphids for egg deposition, and embryos were collected in batch from leaves heavily infested with aphids. For subsequent analyses, we removed all reads that matched published *A. pisum *sequences (mRNA + genomic, NCBI, 2009-07-13) [68] with 95% or higher identity. Removal of *A. pisum *sequences reduced the total sequence data by about 26% to 158.1 MB and the number of reads from each library by about 22%, resulting in 325,200 reads from the maternal library with a mean read length of 228 nt, and 311,906 reads from the zygotic library with a mean read length of 269 nt. The distribution of read lengths displayed two peaks, one at about 400 nt (360 nt for reads from the zygotic library) and one at less than 100 nt (Additional file 1C,D). The peak at 400 nt corresponded to the expected mean length using the Titanium chemistry. The peak slightly below 100 nt presumably resulted from our cDNA preparation protocol, which had not yet been optimized for the Titanium chemistry at the time of library preparation and lacked, for example, any additional size exclusion steps following gel electrophoreses.

All reads have been deposited as the *E. balteatus *transcriptome at the NCBI Sequence Read Archive (SRA, SRP005289). Assembly was based on combined SFF sequence files of the maternal and zygotic libraries using Newbler Assembler (software release 2.3). Newbler parameters were default except: minimal identity of 90% in overlaps (-mi 90), overlaps to be at least 30 nt in length (-ml 30), minimal length of contigs to be 100 nt (-l 100). Newbler was run as cDNA assembly (-cdna), which includes processing of contigs that are found to be variants of the same transcript into distinct isotigs. From a total of 637,080 reads, 544,776 reads (85.5%) were assembled (fully assembled reads: 432,160 reads, 67.8%; partially assembled reads: 112,616 reads, 17.7%). The remaining reads were singletons (56,625 reads; 8.9%) or excluded as either originating from repeat regions (221 reads; 0.03%), outliers (23,060 reads; 3.6%), or too short (< 50 base pairs: 12,398 reads; 1.9%) (see Additional file 4 for comparison with Newbler 2.5.3).

### Sequence Annotation

Reported isotigs (12,296; 12.9 MB) and singletons of at least 100 nt length (26,862; 7.1 MB; identified from 454ReadStatus.txt) were combined, and reciprocal BLAST searches of the *E. balteatus *transcriptome were carried out against the translated transcriptome of *D. melanogaster *(dmel-all-translation-r5.29.faa, flybase.org) using blastx (*E. balteatus *query against *D. melanogaster *database) and tblastn (*D. melanogaster *query against *E. balteatus *database). Annotation was performed with an e-value threshold of 10 to screen for all putative orthologues ("no cutoff") and with an e-value threshold of 1e-10 to obtain a conservative list of high confidence orthologues ("1e-10") (see Additional file 5 for comparison of annotation based on assemblies with Newbler 2.3 and Newbler 2.5.3). Reciprocal hits of *E. balteatus *with *D. melanogaster *were assigned with the same flybase *D. melanogaster *CG identifiers. Gene ontology terms were assigned based on the current *D. melanogaster *gene ontology, with the exception of 'transcription factor - strict' and 'transcription factor - putative', which were based on a curated list of genes of known and putative gene specific transcription factors [30].

### Sequence Coverage

To determine coverage levels for individual published *E. balteatus *genes, associated reads of the maternal and zygotic library sequences were identified by BLAST search (blastn) and assembled with the published sequence using CAP3 with standard parameters [69]. Coverage levels were then calculated for each nucleotide position of the published gene sequences. Fold-coverage in the maternal library was used to approximate maternal expression levels of all annotated *E. balteatus *genes. The assembled *E. balteatus *transcriptome (isotigs and singletons) was blasted against all maternal reads (blastn). Coverage of annotated transcripts was then calculated from all reads that matched with at least 95% identity and over the length of at least 50 nt. To approximate levels of gene expression, coverage of each gene was divided by its sequence length and the total RNAseq data of the maternal library (74 MB). Fold-coverage in the 0-2 hr time point of the *D. melanogaster *development transcriptome was used to approximate maternal expression levels of *D. melanogaster *genes [67]. Expression data of *D. melanogaster *genes in 0-2 hrs old embryos was downloaded from modEncode as coverage data mapped onto the entire genome (BC1_plus.wig, BC1_minus.wig). Expression of gene transcripts was retrieved from this genomic map by extracting coverage information of all exons for each annotated gene (BDGP/dm3). To account for potentially mis-annotated exon-intron structures of computationally predicted genes, gene expression was approximated by the coverage of the most strongly expressed exon longer than 500 nt, or by the coverage of the most strongly expressed transcript variant, whichever was higher. To approximate levels of gene expression, coverage of each gene was divided by its sequence length and the total RNAseq data of the 0-2 hr time point (3.4 GB, SRP001696).

### Gene Discovery of BMP Signaling Components

A list of *D. melanogaster *and *Tribolium castaneum *BMP signaling compounds was matched against assembled and unassembled *E. balteatus *transcriptome data (tblastn, e-value of 0.001). Identified *E. balteatus *sequences were assembled in Sequencher, assemblies were manually corrected for ORF frame shifts by alignment with *D. melanogaster *protein sequence, and sequence orthology was confirmed by reciprocal blast against a *D. melanogaster *database. To exhaustively search for putative duplicates specific to the *E. balteatus *lineage, blast searches were repeated using lower cut-offs (increasingly higher e-values) until all newly identified and assembled *E. balteatus *reads matched a clearly non-orthologous sequence in available insect protein databases. All *T. castaneum *sequences were retrieved from BeetleBase (ftp://bioinformatics.ksu.edu/pub/BeetleBase/latest/Sequences/Tribolium_Official_Gene_Sequences/mRNA.fa).

### Phylogenetic Gene Trees

Protein alignments were created using the Clustal algorithm with standard parameters (MegAlign). When more than half of the aligned sequences carried a gap at a given position, these positions were removed from the alignment. The amino acid substitution model was estimated using AIC in ProtTest [70]; maximum likelihood trees were calculated using PhyML [71]. Bootstrap values were based on 1000 replicas. Trees were plotted with drawtree (Phylip package) [72] and the newick-utils package [73].

Custom scripts (Perl, R) were used to automate blast searches and evaluation, calculate *E. balteatus *and *D. melanogaster *gene coverage, and prune sequence alignments. Scripts are available on request. Plots were prepared with gnuplot and finished with Freehand. Assembly, blast searches, and bootstrap analysis were computed on the computer cluster of the Department of Ecology & Evolution at the 	University of Chicago (http://biocomputing.uchicago.edu).

## Authors' contributions

SL designed the research, performed the bioinformatic analyses and wrote the manuscript. DAA prepared the cDNA libraries for 454 sequencing, FM and MHD performed 454 sequencing. USO designed the research, analyzed the data, wrote the manuscript and obtained funding. All authors read and approved the final manuscript.

## Supplementary Material

Additional file 1 **Normalization and 454 sequencing of transcriptome libraries**. (**A**) Normalization of library of 0-0.5 hrs old embryos. (**B**) Normalization of library of 3-6 hrs old embryos. Normalization was assessed by quantitative PCR. Shown are transcript levels of *E. balteatus alpha tubulin *(*Eba-tub*) relative to *E. balteatus *orthologues of *hunchback *(*Eba-hb*) and *caudal *(*Eba-cad*). Bar heights indicate transcript levels, colors indicate levels in the non-normalized cDNA library (blue), and after normalization with 1 unit DSN (red), 2 units DSN (orange), and 4 units DSN (yellow). (**C**) Distribution of read lengths of library from 0-0.5 hrs old embryos. (**D**) Distribution of read lengths of library from 3-6 hrs old embryos. Read length (x-axis) is shown as a function of number of reads (y-axis) before (grey graph) and after removal of pea aphid sequence contaminations (black graph). Click here for file

Additional file 2 **Coverage of previously identified *E. balteatus *embryonic patterning genes in the libraries of 0-0.5 hrs old embryos (maternal) and 3-6 hrs old embryos (zygotic)**. Click here for file

Additional file 3 **Expression of *Eba-otd *in ovarian follicles and early embryos**. (**A-C**) *Eba-otd *in situ hybridizations of ovarian follicles (A,B) and an early embryo (C). Anterior is left, dorsal is up. (**D**) Increase of zygotic expression levels relative to maternal expression levels measured for *Eba-otd, Eba-cad*, and *Eba-hb *using qPCR (orange bars) and transcriptome coverage (grey bars). qPCR data are based on the average of two independent PCRs, each of which run in triplicate. Bars indicate variance between samples. Click here for file

Additional file 4 **Schematic overview of TGF-β signaling in *D. melanogaster***. TGF-β ligands bind as dimers to a transmembrane receptor complex, which activates signal transducing protein that translocates to the nucleus to regulate gene activity (modified after [74]). Components of the BMP signaling cascade are shown in red, components of the Activin-β signaling cascade in blue, shared components in purple. In the case of generalized BMP signaling, extraecellular ligands (Dpp, Scw, Gbb) are regulated by BMP antagonists Sog and Tsg/Cv/Srw(?), and metalloprotease activity of Tld/Tok, which cleaves Sog and frees BMP. Additional BMP specific regulators are transmembrane protein CV-2, which directly binds BMPs, and membrane associated protein Kek5. Active BMPs signal through the receptor complex composed of Tkv, Sax and Put, which phophorylates Mad. Phophorylated Mad recruits Medea and translocates to the nucleus. For abbreviations, see legend to Figure 3. Click here for file

Additional file 5 **Comparison of sequence annotation following de novo transcriptome assemblies produced by Newbler v2.3 and Newbler v2.5.3**. During manuscript preparation, a new version of Newbler became available (v2.5). To address the concern of a potential underperformance of Newbler v2.3 in the case of our data set, we repeated the assembly and annotation with the latest available Newbler assembler (v2.5.3) and compared the results to the assembly with Newbler v2.3. We found the total number of assembled bases with Newbler v2.5.3 (10.5 Mb) decreased by almost 20% when compared with the number of assembled bases with Newbler v2.3 (12.9 Mb), suggesting that for our dataset Newbler v2.5.3 performed with more stringent assembly conditions. The observed differences did not affect our identification of BMP signaling components in *E. balteatus *as these genes were all identified by Blast and subsequent manual assembly. Click here for file
