# Supplementary figures and images for: BMP signaling components in embryonic transcriptomes of the hover fly Episyrphus balteatus (Syrphidae)
Source: BMC Genomics. 2011 May 31;12:278. doi: 10.1186/1471-2164-12-278 (PMC3224130; doi:10.1186/1471-2164-12-278)

**A**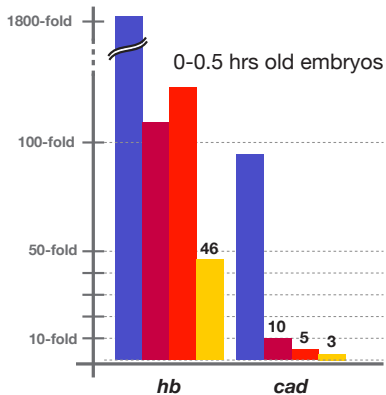**B**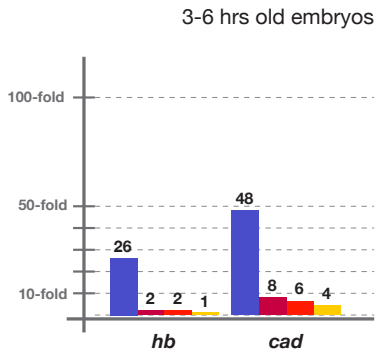**C**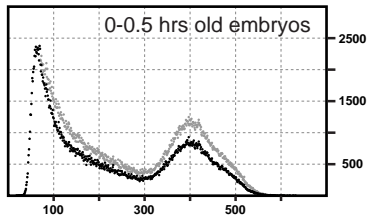**D**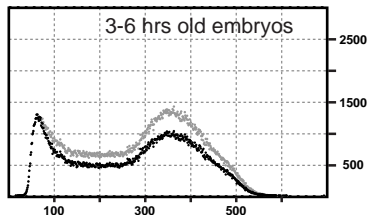

Supplement: Additional file 1 — Normalization and 454 sequencing of transcriptome libraries. (A) Normalization of library of 0-0.5 hrs old embryos. (B) Normalization of library of 3-6 hrs old embryos. Normalization was assessed by quantitative PCR. Shown are transcript levels of E. balteatus alpha tubulin (Eba-tub) relative to E. balteatus orthologues of hunchback (Eba-hb) and caudal (Eba-cad). Bar heights indicate transcript levels, colors indicate levels in the non-normalized cDNA library (blue), and after normalization with 1 unit DSN (red), 2 units DSN (orange), and 4 units DSN (yellow). (C) Distribution of read lengths of library from 0-0.5 hrs old embryos. (D) Distribution of read lengths of library from 3-6 hrs old embryos. Read length (x-axis) is shown as a function of number of reads (y-axis) before (grey graph) and after removal of pea aphid sequence contaminations (black graph). [file 1471-2164-12-278-S1.PDF]

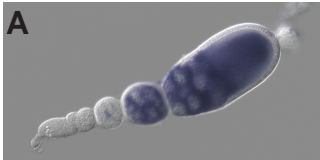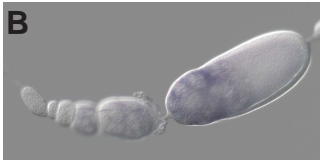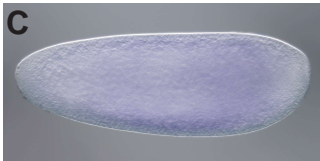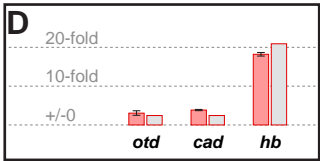

Supplement: Additional file 3 — Expression of Eba-otd in ovarian follicles and early embryos. (A-C) Eba-otd in situ hybridizations of ovarian follicles (A,B) and an early embryo (C). Anterior is left, dorsal is up. (D) Increase of zygotic expression levels relative to maternal expression levels measured for Eba-otd, Eba-cad, and Eba-hb using qPCR (orange bars) and transcriptome coverage (grey bars). qPCR data are based on the average of two independent PCRs, each of which run in triplicate. Bars indicate variance between samples. [file 1471-2164-12-278-S3.PDF]

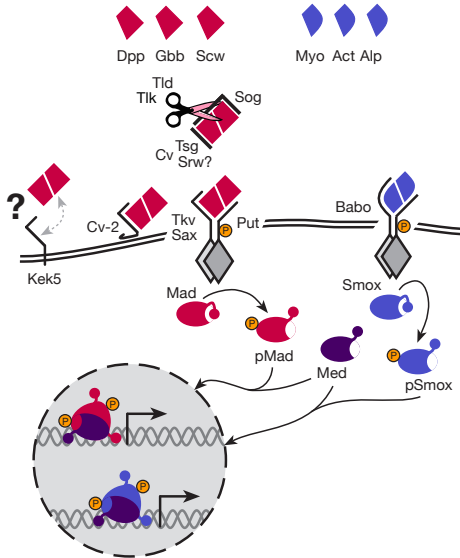

Supplement: Additional file 4 — Schematic overview of TGF-β signaling in D. melanogaster. TGF-β ligands bind as dimers to a transmembrane receptor complex, which activates signal transducing protein that translocates to the nucleus to regulate gene activity (modified after [74]). Components of the BMP signaling cascade are shown in red, components of the Activin-β signaling cascade in blue, shared components in purple. In the case of generalized BMP signaling, extraecellular ligands (Dpp, Scw, Gbb) are regulated by BMP antagonists Sog and Tsg/Cv/Srw(?), and metalloprotease activity of Tld/Tok, which cleaves Sog and frees BMP. Additional BMP specific regulators are transmembrane protein CV-2, which directly binds BMPs, and membrane associated protein Kek5. Active BMPs signal through the receptor complex composed of Tkv, Sax and Put, which phophorylates Mad. Phophorylated Mad recruits Medea and translocates to the nucleus. For abbreviations, see legend to Figure 3. [file 1471-2164-12-278-S4.PDF]
