# Supplementary material for: BMP signaling components in embryonic transcriptomes of the hover fly Episyrphus balteatus (Syrphidae)
Source: BMC Genomics. 2011 May 31;12:278. doi: 10.1186/1471-2164-12-278 (PMC3224130; doi:10.1186/1471-2164-12-278)
Supplement: Additional file 2 — Coverage of previously identified E. balteatus embryonic patterning genes in the libraries of 0-0.5 hrs old embryos (maternal) and 3-6 hrs old embryos (zygotic). [file 1471-2164-12-278-S2.PDF]

		Total	ORF	5'UTR	3'UTR
Eba-bcd	%CG		0.41	-	0.22
	length (nt)		1023	0	734
maternal	coverage	5.63	9.67	-	-
zygotic	coverage	7.64	12.90	-	0.31
Eba-hb	%CG		0.43	0.28	0.24
	length (nt)		2118	601	187
maternal	coverage	0.55	0.24	1.82	-
zygotic	coverage	10.41	13.75	1.86	-
Eba-nos	%CG		0.36	0.20	0.22
	length (nt)		672	174	597
maternal	coverage	11.62	21.68	8.62	1.17
zygotic	coverage	1.09	1.68	0.77	0.52
Eba-cad	%CG		0.45	0.27	0.26
	length (nt)		1110	197	288
maternal	coverage	5.96	7.83	0.39	2.52
zygotic	coverage	9.32	12.35	2.89	2.03
Eba-eve	%CG		0.50	-	0.23
	length (nt)		688	0	290
maternal	coverage	-	-	-	-
zygotic	coverage	10.20	13.38	-	2.66
Eba-gt	%CG		0.42	0.30	0.26
	length (nt)		1164	153	168
maternal	coverage	-	-	-	-
zygotic	coverage	5.69	6.26	6.07	1.42
Eba-h	%CG		0.47	-	0.32
	length (nt)		906	0	621
maternal	coverage	-	-	-	-
zygotic	coverage	15.40	23.82	-	3.11
Eba-hkb	%CG		0.43	-	-
	length (nt)		631	0	0
maternal	coverage	-	-	-	-
zygotic	coverage	0.73	0.73	-	-
Eba-kni	%CG		0.45	0.28	0.12
	length (nt)		1479	387	233
maternal	coverage	-	-	-	-
zygotic	coverage	3.48	4.82	0.45	-

Eba-Kr	%CG		0.47	0.29	0.18
	length (nt)		1518	712	679
maternal	coverage	-	-	-	-
zygotic	coverage	10.33	18.70	1.85	0.49
Eba-otd	%CG		0.48	0.32	0.27
	length (nt)		987	522	397
maternal	coverage	2.27	2.01	1.14	4.41
zygotic	coverage	5.18	8.01	0.47	4.31
Eba-tll	%CG		0.37	0.28	0.23
	length (nt)		1257	97	311
maternal	coverage	-	-	-	-
zygotic	coverage	0.04	0.06	-	-
**Eba-tor**	%CG		0.37	-	0.18
	length (nt)		1555	0	274
maternal	coverage	2.18	2.57	-	-
zygotic	coverage	10.31	12.12	-	-
Eba-zen	%CG		0.41	0.28	0.09
	length (nt)		993	123	78
maternal	coverage	-	-	-	-
zygotic	coverage	2.34	2.63	1.48	-
mean %CG			0.43	0.19	0.22
		120.3	175.2		
total coverage		6	2	27.81	22.95
mean coverage		8.60	12.52	1.99	1.64
