# Supplementary material for: BMP signaling components in embryonic transcriptomes of the hover fly Episyrphus balteatus (Syrphidae)
Source: BMC Genomics. 2011 May 31;12:278. doi: 10.1186/1471-2164-12-278 (PMC3224130; doi:10.1186/1471-2164-12-278)
Supplement: Additional file 5 — Comparison of sequence annotation following de novo transcriptome assemblies produced by Newbler v2.3 and Newbler v2.5.3. During manuscript preparation, a new version of Newbler became available (v2.5). To address the concern of a potential underperformance of Newbler v2.3 in the case of our data set, we repeated the assembly and annotation with the latest available Newbler assembler (v2.5.3) and compared the results to the assembly with Newbler v2.3. We found the total number of assembled bases with Newbler v2.5.3 (10.5 Mb) decreased by almost 20% when compared with the number of assembled bases with Newbler v2.3 (12.9 Mb), suggesting that for our dataset Newbler v2.5.3 performed with more stringent assembly conditions. The observed differences did not affect our identification of BMP signaling components in E. balteatus as these genes were all identified by Blast and subsequent manual assembly. [file 1471-2164-12-278-S5.PDF]

		newbler 2.3		newbler 2.5.3	
Reads	total	637,080		637,076	
assembled	full	432,160	(67.8%)	437,929	(68.7%)
	partial	112,616	(17.7%)	105,230	(16.5%)
excluded	singletons	56,625	(8.9%)	61,263	(9.6%)
	repeat	221	(0.03%)	266	(0.04%)
	outliers	23,060	(3.6%)	19,942	(3.1%)
	too short	12,398	(1.9%)	12,446	(2.0%)
isotigs		12,296	(12,9 Mb)	11,709	(10,5 Mb)
singletons [⩾ 100 nt]		26,862	(7,1 Mb)	16,109	(3,9 Mb)
Annotation 1e-10	total	5,556		5,215	
	specific to assembly	522	(9.4%)	181	(3.5%)
Annotation "no cutoff"	total	6,016		5,598	
	isotigs	4,558	(75.7%)	4,586	(81.9%)
	reads	1,455	(24.2%)	1,012	(18.1%)
	specific to assembly	646	(10.7%)	231	(4.1%)
